# Low-Income Cooperative Living Environments: An Analysis of Universal Design Features That Support Aging in Place

**DOI:** 10.1093/geroni/igaa057.1405

**Published:** 2020-12-16

**Authors:** Migette Kaup, Hannah Richardson, Mikayla Adkins, Brett LaFleur, Sydney Tucker, Lauren Tines

**Affiliations:** 1 Kansas State University, Manhattan, Kansas, United States; 2 StudioSIX, Austin, Texas, United States

## Abstract

Affordable housing for low-income older adults is in significant demand in the United States. Housing options available, however may not recognize how design can support social infrastructure in addition to basic physical accessibility. The aim of this research was to provide an analysis of the efficacy of the planning and design principles applied to a recently constructed cooperative living home. The goal was assess the universal design (UD) of features that supported aging in place, and, to inform the planning of future projects for the community. A post-occupancy evaluation process was conducted at the home, and all five residents consented to structured focus group discussions as well as individual interviews regarding their specific experiences living in the home. An environmental assessment was completed through detailed field notes, measurements, and photo documentation. An analysis of specific features of each room of the home was then assessed by the seven UD principles. Results revealed the specific features of each space that contributed to the completion (or the limitations) of basic activities of daily living as well as instrumental activities of daily living. These assessments were validated through feedback provided by the users. Many features of the home were found to be positive and supportive of equitable use by all residents. Other design attributes that could be improved were related to size and space for approach and use, and providing flexibility in use. Specific design changes based on resident input, such as kitchen function, storage safety, and lighting details, are highlighted and discussed.

